# Supplementary material for: Prescription of Opioid Analgesics for Chronic Non-Cancer Pain in Germany despite Contraindications: Administrative Claims Data Analysis
Source: Int J Environ Res Public Health. 2024 Feb 5;21(2):180. doi: 10.3390/ijerph21020180 (PMC10888146; doi:10.3390/ijerph21020180)
Supplement: Supplementary file 1 [file ijerph-21-00180-s001.zip › ijerph-2793585-supplementary.pdf]

Table S1. Indications and related tracer diagnosis (ICD-10-GM) for opioid analgesics therapy based on the German Guideline for long-term opioid analgesics therapy in chronic non-tumor related pain [1].

No.	Indication based on German guideline [1]	Tracer diagnosis - ICD-10-GM
1	Chronic back pain	M42.16-M42.19, M42.90, M42.96-99, M43.0, M43.1, M47.26, M47.27, M47.29, M47.86, M47.87, M47.88, M47.99, M48.06, M48.2, M54.16, M54.5, M55.3, M.99.33; M99.43, M99.53
2	Chronic arthrosis pain	M15-M19
3	Diabetic polyneuropathy	G63.2 (and E10-E14)
4	Postzoster neuralgia	B02.2, G53.0
5	Phantom Pain	G54.6
6	Pain after spinal cord injury	S14.0, S14.1, S14.7, S19.80, S24.0, S24.1, S24.2, S24.7, S29.8, S34.0, S34.1, S34.2, S34.3, S34.7, S39.81
7	Painful radiculopathy	M54.1
8	Polyneuropathy of etiology other than diabetes	G60-G64 (without G63.2)
9	Rheumatoid arthritis with persistent pain	M06.-
10	Restless legs syndrome	G25.81
11	Parkinson syndrome	G20, G21
12	Fibromyalgia Syndrome	M79.70
13	Chronic pain in manifest osteoporosis (vertebral body fractures)	M80.08, M80.18, M80.28, M80.38, M80.48, M80.58, M80.88, M80.98
14	Chronic pain associated with inflammatory rheumatic diseases other than rheumatoid arthritis (e.g., systemic lupus erythematosus, spondyloarthritis)	M32, M45-M49
15	Chronic extremity pain in ischemic and inflammatory arterial occlusive disease	I70, I73, I77, I79
16	Chronic pain in decubitus grade 3 and 4	L89.2-, L89.3-
17	Chronic fixed contracture pain in patients in need of long-term care	M24.5, M67.1
18	Central (cerebral) neuropathic pain (e.g., after thalamic infarction, multiple sclerosis)	D33.-, D43.-, G04.8, G04.9, G05.-, G06.-, G07, G35.-, G37.3, G37.4, I60.-, I62.-, I63.-, Q27.3, Q28.-, S06.-,
19	Secondary headache and central (cerebral) neuropathic pain (e.g., after thalamic infarction, multiple sclerosis)	(D33.-, D43.-, G04.8, G04.9, G05.-, G06.-, G07, G35.-, G37.3, G37.4, I60.-, I62.-, I63.-, Q27.3, Q28.-, S06.-) and G44.8
20	Chronic complex regional pain syndrome type I and II	G56.4/G57.8/ M89.0 (until 2018), G90.5-G90.7 (since 2019),
21	Traumatic trigeminal neuropathy	G50.9
22	Chronic lower abdominal pain in women with marked adhesions and/or multilocular endometriosis	N73.6, N80.-

Table S2. Detailed logistic regression models (models A and models B) on contraindication groups.

Models A

Logistic regression	Number of obs = 113,476
	LR chi2(14) = 5573.63
	Prob > chi2 = 0.0000
Log likelihood = -30640.814	Pseudo R2 = 0.0834

primary headache	Odds ratio	Std. err.	z	P>|z|	[95% conf.	interval]
female	2.197647	.0635586	27.23	0.000	2.076539	2.325818
age group (ref. 70-89 years)						
18-49 years	3.71238	.1316796	36.98	0.000	3.46306	3.97965
50-69 years	2.347369	.0580213	34.52	0.000	2.23636	2.463888
>89 years	.3892202	.0292834	-12.54	0.000	.3358569	.4510623
region (ref. east)						
north	1.001944	.0345695	0.06	0.955	.9364286	1.072042
west	1.035202	.0327126	1.09	0.274	.9730311	1.101344
south	1.005931	.0357844	0.17	0.868	.938184	1.07857
history of >3 quarters of OA prescriptions prior to inclusion	1.287482	.0283414	11.48	0.000	1.233115	1.344245
outpatient pain therapy	1.611315	.0418818	18.35	0.000	1.531284	1.695528
IMPT (inpatient)	1.37599	.0671248	6.54	0.000	1.250522	1.514047
outpatient psychotherapy	1.521783	.0691386	9.24	0.000	1.392132	1.663509
outpatient psychosomatic primary care	1.569469	.0360713	19.61	0.000	1.50034	1.641784
ICD coding F11	1.143085	.0586725	2.61	0.009	1.033685	1.264065
indication	1.247883	.0338173	8.17	0.000	1.183332	1.315956
_cons	.0171873	.0008094	-86.28	0.000	.0156718	.0188493

Logistic regression	Number of obs = 113,476
	LR chi2(14) = 4078.25
	Prob > chi2 = 0.0000
Log likelihood = -18906.877	Pseudo R2 = 0.0974

pain in somatoform disorders	Odds ratio	Std. err.	z	P>|z|	[95% conf.	interval]
female	1.279574	.0456547	6.91	0.000	1.193149	1.372258
age group (ref. 70-89 years)						
18-49 years	1.803808	.0900189	11.82	0.000	1.635728	1.989159
50-69 years	1.603292	.0534368	14.16	0.000	1.501905	1.711522
>89 years	.6455738	.0561041	-5.04	0.000	.544467	.7654561
region (ref. east)						
north	.8288817	.0375933	-4.14	0.000	.7583802	.9059372
west	.799864	.0334186	-5.34	0.000	.7369748	.8681198
south	1.017585	.0455452	0.39	0.697	.9321216	1.110885
history of >3 quarters of OA prescriptions prior to inclusion	1.465243	.0438354	12.77	0.000	1.381797	1.553727
outpatient pain therapy	2.029267	.0665122	21.59	0.000	1.903004	2.163907
IMPT (inpatient)	1.545011	.0880654	7.63	0.000	1.381699	1.727627
outpatient psychotherapy	2.218085	.1168589	15.12	0.000	2.000475	2.459367
outpatient psychosomatic primary care	2.611364	.084399	29.70	0.000	2.451075	2.782135
ICD coding F11	1.713754	.0975525	9.46	0.000	1.532834	1.916027
indication	1.344361	.0521077	7.63	0.000	1.246015	1.45047
_cons	.0107581	.0006679	-73.01	0.000	.0095256	.01215

Logistic regression | Number of obs = 113,476
LR chi2(14) = 429.54
Prob > chi2 = 0.0000
Log likelihood = -6283.1824 | Pseudo R2 = 0.0331

chronic pancreatitis	Odds ratio	Std. err.	z	P>|z|	[95% conf.	interval]
female	.4710428	.0288344	-12.30	0.000	.4177869	.5310872
age group (ref. 70-89 years)						
18-49 years	1.651034	.1720311	4.81	0.000	1.34606	2.025106
50-69 years	1.727476	.1156813	8.16	0.000	1.514994	1.96976
>89 years	.8428243	.1293441	-1.11	0.265	.623889	1.138588
region (ref. east)						
north	.6351831	.054901	-5.25	0.000	.5361999	.7524387
west	.6501441	.0503784	-5.56	0.000	.5585366	.7567763
south	.5536356	.0513816	-6.37	0.000	.4615578	.6640825
history of >3 quarters of OA prescriptions prior to inclusion	1.163602	.0694342	2.54	0.011	1.03517	1.307968
outpatient pain therapy	.9960486	.0798067	-0.05	0.961	.8512938	1.165418
IMPT (inpatient)	.7916834	.1390312	-1.33	0.183	.5611359	1.116953
outpatient psychotherapy	.8490704	.1443601	-0.96	0.336	.6084448	1.184858
outpatient psychosomatic primary care	1.274429	.0802591	3.85	0.000	1.126445	1.441854
ICD coding F11	1.839579	.2211496	5.07	0.000	1.453413	2.328347
indication	.8231409	.0549987	-2.91	0.004	.7221054	.938313
_cons	.0183664	.0018469	-39.75	0.000	.0150809	.0223675

Logistic regression | Number of obs = 113,476
LR chi2(14) = 500.02
Prob > chi2 = 0.0000
Log likelihood = -10783.414 | Pseudo R2 = 0.0227

chronic inflammatory bowel disease	Odds ratio	Std. err.	z	P>|z|	[95% conf.	interval]
female	1.301869	.0668262	5.14	0.000	1.177265	1.439661
age group (ref. 70-89 years)						
18-49 years	2.76508	.1910423	14.72	0.000	2.414889	3.166052
50-69 years	1.856355	.0906547	12.67	0.000	1.686913	2.042816
>89 years	.5687432	.0686348	-4.68	0.000	.4489469	.7205057
region (ref. east)						
north	.9552998	.065912	-0.66	0.507	.8344687	1.093627
west	1.113119	.0691893	1.72	0.085	.9854457	1.257334
south	.9450651	.0674687	-0.79	0.429	.8216635	1.087
history of >3 quarters of OA prescriptions prior to inclusion	1.296731	.0561555	6.00	0.000	1.19121	1.4116
outpatient pain therapy	.9805474	.0562038	-0.34	0.732	.8763522	1.097131
IMPT (inpatient)	.9345896	.1093746	-0.58	0.563	.7430281	1.175538
outpatient psychotherapy	1.050682	.1091311	0.48	0.634	.8571553	1.287902
outpatient psychosomatic primary care	1.27148	.0578447	5.28	0.000	1.163014	1.390062
ICD coding F11	1.206903	.1232201	1.84	0.065	.9880255	1.474269
indication	1.005395	.0503832	0.11	0.914	.9113402	1.109156
_cons	.009339	.0008019	-54.43	0.000	.0078923	.0110507

Logistic regression | Number of obs = 113,476
LR chi2(13) = 1107.40
Prob > chi2 = 0.0000
Log likelihood = -3923.1475 | Pseudo R2 = 0.1237

harmful use of opioids	Odds ratio	Std. err.	z	P>|z|	[95% conf.	interval]
female	.5960422	.0463781	-6.65	0.000	.5117348	.6942392
age group (ref. 70-89 years)						
18-49 years	5.014339	.5408245	14.95	0.000	4.058886	6.194704
50-69 years	2.100912	.1957506	7.97	0.000	1.750241	2.521842
>89 years	.4322932	.1407354	-2.58	0.010	.2283837	.8182607
region (ref. east)						
north	1.722543	.220868	4.24	0.000	1.339761	2.21469
west	1.367607	.1690392	2.53	0.011	1.073374	1.742496
south	1.7659	.2289878	4.39	0.000	1.369585	2.276895
history of >3 quarters of OA prescriptions prior to inclusion	2.68433	.2202072	12.04	0.000	2.285641	3.152562
outpatient pain therapy	2.065538	.1713147	8.75	0.000	1.755638	2.430141
IMPT (inpatient)	4.638626	.4732674	15.04	0.000	3.797899	5.665462
outpatient psychotherapy	.7758411	.1223696	-1.61	0.108	.5695305	1.056887
outpatient psychosomatic primary care	1.924719	.1569774	8.03	0.000	1.64038	2.258344
indication	.9685638	.0858703	-0.36	0.719	.8140724	1.152374
_cons	.0011158	.0001844	-41.15	0.000	.0008072	.0015425

Logistic regression | Number of obs = 113,476
LR chi2(13) = 2584.44
Prob > chi2 = 0.0000
Log likelihood = -9986.1678 | Pseudo R2 = 0.1146

mental and behavioral disorders caused by opioids	Odds ratio	Std. err.	z	P>|z|	[95% conf.	interval]
female	.6706052	.0306899	-8.73	0.000	.613073	.7335364
age group (ref. 70-89 years)						
18-49 years	3.860366	.244617	21.32	0.000	3.409502	4.37085
50-69 years	1.865922	.0952196	12.22	0.000	1.688324	2.062201
>89 years	.4027846	.0702042	-5.22	0.000	.2862271	.5668067
region (ref. east)						
north	1.057072	.0684671	0.86	0.391	.9310475	1.200155
west	.8330283	.0518886	-2.93	0.003	.7372914	.9411966
south	.9193996	.0627538	-1.23	0.218	.8042766	1.051001
history of >3 quarters of OA prescriptions prior to inclusion	2.924691	.1393624	22.52	0.000	2.663912	3.210997
outpatient pain therapy	1.533604	.0757418	8.66	0.000	1.392111	1.689478
IMPT (inpatient)	3.720425	.2504826	19.51	0.000	3.2605	4.245226
outpatient psychotherapy	.9533821	.0872863	-0.52	0.602	.7967751	1.14077
outpatient psychosomatic primary care	2.354245	.1099814	18.33	0.000	2.14826	2.579982
indication	.9112611	.0462942	-1.83	0.067	.824897	1.006667
_cons	.005923	.000517	-58.76	0.000	.0049916	.0070282

Logistic regression | Number of obs = 113,476
LR chi2(14) = 4177.04
Prob > chi2 = 0.0000
Log likelihood = -28700.064 | Pseudo R2 = 0.0678

severe mood disorder	Odds ratio	Std. err.	z	P>|z|	[95% conf.	interval]
female	1.221607	.0336855	7.26	0.000	1.157337	1.289446
age group (ref. 70-89 years)						

18-49 years	1.633945	.0656351	12.22	0.000	1.510237	1.767787
50-69 years	1.541079	.0401367	16.61	0.000	1.464387	1.621788
>89 years	.7788542	.043845	-4.44	0.000	.6974907	.869709
region (ref. east)						
north	1.125701	.0439162	3.04	0.002	1.042835	1.215151
west	1.466551	.0517773	10.85	0.000	1.368501	1.571626
south	1.355742	.052502	7.86	0.000	1.256648	1.46265
history of >3 quarters of OA prescriptions prior to inclusion	1.214747	.0280664	8.42	0.000	1.160965	1.271021
outpatient pain therapy	1.136281	.0327336	4.43	0.000	1.073902	1.202283
IMPT (inpatient)	1.332218	.0685634	5.57	0.000	1.204392	1.473612
outpatient psychotherapy	3.164023	.1360514	26.79	0.000	2.908295	3.442238
outpatient psychosomatic primary care	2.232758	.0544864	32.92	0.000	2.12848	2.342144
ICD coding F11	2.066997	.0974976	15.39	0.000	1.884472	2.267201
indication	1.161225	.0327156	5.31	0.000	1.098842	1.22715
_cons	.0224123	.0010893	-78.14	0.000	.0203758	.0246524

Logistic regression	Number of obs	= 113,476
	LR chi2(14)	= 273.3
	Prob > chi2	= 0.0000
Log likelihood = -4254.6195	Pseudo R2	= 0.0311

suicidality	Odds ratio	Std. err.	z	P>|z|	[95% conf.	interval]
female	1.18386	.1077885	1.85	0.064	.9903753	1.415145
age group (ref. 70-89 years)						
18-49 years	1.503315	.1902438	3.22	0.001	1.173089	1.9265
50-69 years	1.161572	.0999953	1.74	0.082	.9812267	1.375064
>89 years	.8856052	.1515108	-0.71	0.478	.6333083	1.238412
region (ref. east)						
north	1.378382	.171279	2.58	0.010	1.080435	1.758493
west	1.322068	.1568599	2.35	0.019	1.047758	1.668194
south	1.23906	.1593728	1.67	0.096	.9629585	1.594327
history of >3 quarters of OA prescriptions prior to inclusion	1.174378	.0886877	2.13	0.033	1.012806	1.361726
outpatient pain therapy	.9675921	.0940564	-0.34	0.735	.7997419	1.170671
IMPT (inpatient)	1.018909	.1853205	0.10	0.918	.7133752	1.455302
outpatient psychotherapy	1.078647	.1902285	0.43	0.668	.7634189	1.524039
outpatient psychosomatic primary care	2.720788	.2220132	12.27	0.000	2.318663	3.192654
ICD coding F11	2.144806	.2995634	5.46	0.000	1.631178	2.820166
indication	1.168234	.1086999	1.67	0.095	.9734835	1.401945
_cons	.0019743	.0003173	-38.74	0.000	.0014408	.0027054

Models B

Logistic regression	Number of obs	= 113,476
	LR chi2(13)	= 1622.93
	Prob > chi2	= 0.0000
Log likelihood = -9567.7396	Pseudo R2	= 0.0782

primary headache	Odds ratio	Std. err.	z	P>|z|	[95% conf.	interval]
female	1.834905	.1023816	10.88	0.000	1.644824	2.046953
age group (ref. 70-89 years)						
18-49 years	9.49829	.6134405	34.86	0.000	8.368953	10.78002
50-69 years	3.357846	.1910862	21.29	0.000	3.003455	3.754053
>89 years	.4829825	.0806259	-4.36	0.000	.348207	.6699238

region (ref. east)						
north	1.086947	.0808139	1.12	0.262	.9395548	1.257462
west	1.113233	.0765211	1.56	0.119	.9729185	1.273784
south	1.361543	.1003256	4.19	0.000	1.178448	1.573085
history of >3 quarters of OA prescriptions prior to inclusion	1.078066	.0486621	1.67	0.096	.9867877	1.177788
outpatient pain therapy	.8153957	.0490897	-3.39	0.001	.7246413	.9175161
IMPT (inpatient)	1.133068	.122116	1.16	0.246	.9173143	1.399568
outpatient psychotherapy	1.19416	.1085891	1.95	0.051	.9992169	1.427135
outpatient psychosomatic primary care	1.180299	.0558921	3.50	0.000	1.075682	1.29509
ICD coding F11	1.351554	.1279627	3.18	0.001	1.122647	1.627135
_cons	.0040961	.0003626	-62.11	0.000	.0034437	.0048721

Logistic regression

Number of obs = 113,476
LR chi2(13) = 620.47
Prob > chi2 = 0.0000
Log likelihood = -5016.5621
Pseudo R2 = 0.0582

pain in somatoform disorders	Odds ratio	Std. err.	z	P>|z|	[95% conf.	interval]
female	1.207209	.0947213	2.40	0.016	1.03513	1.407895
age group (ref. 70-89 years)						
18-49 years	4.312843	.4256897	14.81	0.000	3.554247	5.233348
50-69 years	2.165662	.1770469	9.45	0.000	1.845029	2.542014
>89 years	1.074858	.1837301	0.42	0.673	.7688677	1.502625
region (ref. east)						
north	.8868404	.0979961	-1.09	0.277	.7141467	1.101295
west	.9895431	.0993358	-0.10	0.917	.8128051	1.204711
south	1.340435	.1400301	2.80	0.005	1.092255	1.645007
history of >3 quarters of OA prescriptions prior to inclusion	1.484818	.1014089	5.79	0.000	1.298789	1.697493
outpatient pain therapy	1.129285	.0918461	1.49	0.135	.9628851	1.324442
IMPT (inpatient)	1.579736	.2055425	3.51	0.000	1.224145	2.038618
outpatient psychotherapy	1.488003	.1838382	3.22	0.001	1.167993	1.895689
outpatient psychosomatic primary care	1.986317	.1438514	9.48	0.000	1.723469	2.289252
ICD coding F11	1.857694	.2178473	5.28	0.000	1.476236	2.337721
_cons	.0021357	.0002706	-48.53	0.000	.0016661	.0027378

Logistic regression

Number of obs = 113,476
LR chi2(13) = 266.26
Prob > chi2 = 0.0000
Log likelihood = -2193.5053
Pseudo R2 = 0.0572

chronic pancreatitis	Odds ratio	Std. err.	z	P>|z|	[95% conf.	interval]
female	.4546627	.0508259	-7.05	0.000	.3652036	.5660354
age group (ref. 70-89 years)						
18-49 years	5.262512	.8896292	9.82	0.000	3.778307	7.329746
50-69 years	3.359628	.4609953	8.83	0.000	2.567394	4.396325
>89 years	.8124039	.2865299	-0.59	0.556	.4069662	1.621756
region (ref. east)						
north	.9131653	.15377	-0.54	0.590	.6564681	1.270238
west	.9166308	.1419879	-0.56	0.574	.6766152	1.241787
south	.9506904	.1642188	-0.29	0.770	.6776503	1.333744
history of >3 quarters of OA	.8942474	.0982185	-1.02	0.309	.7210528	1.109043

prescriptions prior to inclusion						
outpatient pain therapy	.6785469	.1093529	-2.41	0.016	.4947689	.9305879
IMPT (inpatient)	.5320952	.2072991	-1.62	0.105	.2479532	1.14185
outpatient psychotherapy	.5046467	.1831953	-1.88	0.060	.2477347	1.027988
outpatient psychosomatic primary care	1.14433	.1317971	1.17	0.242	.9130929	1.434127
ICD coding F11	2.101448	.421163	3.71	0.000	1.418812	3.112525
_cons	.002663	.0004856	-32.51	0.000	.0018628	.0038069

Logistic regression — Number of obs = 113,476; LR chi2(13) = 413.51; Prob > chi2 = 0.0000

Log likelihood = -3447.832 — Pseudo R2 = 0.0566

chronic inflammatory bowel disease	Odds ratio	Std. err.	z	P>|z|	[95% conf.	interval]
female	1.473937	.1452863	3.94	0.000	1.214999	1.78806
age group (ref. 70-89 years)						
18-49 years	9.031802	1.091665	18.21	0.000	7.126734	11.44612
50-69 years	3.422352	.3636305	11.58	0.000	2.778965	4.214696
>89 years	.6979244	.1796671	-1.40	0.162	.4213889	1.155936
region (ref. east)						
north	1.290772	.1961224	1.68	0.093	.958333	1.738533
west	1.656904	.2291351	3.65	0.000	1.263524	2.172756
south	1.647222	.2477351	3.32	0.001	1.226691	2.211919
history of >3 quarters of OA prescriptions prior to inclusion	1.131828	.095102	1.47	0.141	.9599713	1.334452
outpatient pain therapy	.5942755	.0756324	-4.09	0.000	.4630809	.7626386
IMPT (inpatient)	.6826166	.1832579	-1.42	0.155	.4033296	1.155297
outpatient psychotherapy	1.004226	.1922216	0.02	0.982	.6900823	1.461377
outpatient psychosomatic primary care	.980756	.0873843	-0.22	0.827	.8236067	1.16789
ICD coding F11	1.39971	.253415	1.86	0.063	.9815892	1.995936
_cons	.0011829	.000202	-39.46	0.000	.0008464	.0016533

Logistic regression — Number of obs = 113,476; LR chi2(11) = 324.76; Prob > chi2 = 0.0000

Log likelihood = -1229.2093 — Pseudo R2 = 0.1167

harmful use of opioids	Odds ratio	Std. err.	z	P>|z|	[95% conf.	interval]
female	.5009221	.0752499	-4.60	0.000	.3731645	.6724191
age group (ref. 70-89 years)2						
1	13.26993	2.823298	12.15	0.000	8.745157	20.13583
2	3.148648	.6627021	5.45	0.000	2.084348	4.756398
region (ref. east)						
north	1.343352	.3626326	1.09	0.274	.7914276	2.280176
west	1.644522	.4086541	2.00	0.045	1.010465	2.676445
south	1.905122	.495973	2.48	0.013	1.143731	3.173377
history of >3 quarters of OA prescriptions prior to inclusion	1.991798	.3040114	4.51	0.000	1.476811	2.686368
outpatient pain therapy	.9908841	.1760525	-0.05	0.959	.6995005	1.403646
IMPT (inpatient)	3.670939	.8091567	5.90	0.000	2.383161	5.654588
outpatient psychotherapy	.6931071	.2222615	-1.14	0.253	.3696937	1.299447
outpatient psychosomatic primary care	2.054081	.3274394	4.52	0.000	1.502894	2.807417

_cons	.0002705	.0000837	-26.56	0.000	.0001475	.0004959

Logistic regression — Number of obs = 113,476; LR chi2(12) = 818.76; Prob > chi2 = 0.0000; Log likelihood = -3297.7815; Pseudo R2 = 0.1104

mental and behavioral disorders caused by opioids	Odds ratio	Std. err.	z	P>|z|	[95% conf.	interval]
female	.5674151	.0484928	-6.63	0.000	.4799046	.6708832
age group (ref. 70-89 years)						
18-49 years	11.47885	1.37231	20.41	0.000	9.081058	14.50977
50-69 years	2.91731	.3382308	9.23	0.000	2.324315	3.661595
>89 years	.5090548	.1866181	-1.84	0.066	.2481512	1.04427
region (ref. east)						
north	1.377985	.1884065	2.34	0.019	1.054055	1.801466
west	1.167042	.1540359	1.17	0.242	.9010274	1.511594
south	1.35554	.1906259	2.16	0.031	1.028987	1.785725
history of >3 quarters of OA prescriptions prior to inclusion	2.082482	.1812175	8.43	0.000	1.755941	2.469749
outpatient pain therapy	.9013071	.0942495	-0.99	0.320	.7342818	1.106325
IMPT (inpatient)	2.197784	.3360584	5.15	0.000	1.628657	2.96579
outpatient psychotherapy	.8049196	.1476463	-1.18	0.237	.5618453	1.153156
outpatient psychosomatic primary care	1.893838	.1677973	7.21	0.000	1.591933	2.252997
_cons	.0012124	.0001996	-40.79	0.000	.000878	.001674

Logistic regression — Number of obs = 113,476; LR chi2(13) = 996.69; Prob > chi2 = 0.0000; Log likelihood = -9156.1611; Pseudo R2 = 0.0516

severe mood disorder	Odds ratio	Std. err.	z	P>|z|	[95% conf.	interval]
female	1.160086	.0635011	2.71	0.007	1.04207	1.291468
age group (ref. 70-89 years)						
18-49 years	3.309038	.2357623	16.80	0.000	2.877765	3.804943
50-69 years	1.992149	.1111774	12.35	0.000	1.78574	2.222416
>89 years	.9778006	.1103796	-0.20	0.842	.7837217	1.219941
region (ref. east)						
north	1.242753	.1073955	2.51	0.012	1.049123	1.472121
west	1.698775	.1330282	6.77	0.000	1.457068	1.980578
south	1.842623	.1527373	7.37	0.000	1.566316	2.167672
history of >3 quarters of OA prescriptions prior to inclusion	1.059164	.0498512	1.22	0.222	.9658285	1.161519
outpatient pain therapy	.6341392	.0424211	-6.81	0.000	.5562153	.7229799
IMPT (inpatient)	1.127899	.1246415	1.09	0.276	.9082509	1.400666
outpatient psychotherapy	2.492091	.2014237	11.30	0.000	2.126989	2.919863
outpatient psychosomatic primary care	1.744456	.0868474	11.18	0.000	1.582279	1.923255
ICD coding F11	2.167536	.1883904	8.90	0.000	1.828035	2.570089
_cons	.0049528	.0004616	-56.95	0.000	.0041259	.0059454

Logistic regression — Number of obs = 113,476; LR chi2(13) = 107.30; Prob > chi2 = 0.0000

Log likelihood = -1130.2055 Pseudo R2 = 0.0453

suicidality	Odds ratio	Std. err.	z	P>|z|	[95% conf.	interval]
female	1.251047	.2379044	1.18	0.239	.8617985	1.816109
age group (ref. 70-89 years)						
18-49 years	3.826535	.8994741	5.71	0.000	2.413906	6.065842
50-69 years	1.98725	.3810476	3.58	0.000	1.364702	2.893791
>89 years	.9016476	.3649263	-0.26	0.798	.4078756	1.993177
region (ref. east)						
north	2.08015	.6175169	2.47	0.014	1.162534	3.722064
west	1.773485	.5174168	1.96	0.050	1.001125	3.141715
south	1.796902	.552448	1.91	0.057	.9836192	3.282629
history of >3 quarters of OA prescriptions prior to inclusion	1.225914	.1996162	1.25	0.211	.8909607	1.686792
outpatient pain therapy	.4416998	.1150348	-3.14	0.002	.2651204	.7358871
IMPT (inpatient)	1.297732	.4920614	0.69	0.492	.6172128	2.728572
outpatient psychotherapy	.5805009	.2693005	-1.17	0.241	.2338415	1.441067
outpatient psychosomatic primary care	2.286307	.3939122	4.80	0.000	1.631096	3.204717
ICD coding F11	2.817993	.733551	3.98	0.000	1.691858	4.693707
_cons	.0002803	.0000956	-23.98	0.000	.0001437	.0005471
